# Lnc-CLSTN2-1:1 Promotes Osteosarcoma Progression by Disrupting Redox Balance through PI3K/AKT Signaling Pathway

**DOI:** 10.7150/jca.91579

**Published:** 2024-01-12

**Authors:** Hao Lin, Xinjian Wei, Junhong Ye, Jiaxian Chen, Jing Huang, Tingrui Wu, Zhenju Chen, Yuming Zeng, Lijiao Peng

**Affiliations:** 1 Department of Orthopedics, Affiliated Hospital of Guangdong Medical University, Zhanjiang, Guangdong,534001, China.; 2 Oncology Hospital, Affiliated Hospital of Guangdong Medical University, Zhanjiang, Guangdong,534001, China.; 3 Department of Orthopedics, Suixi Hospital of Affiliated Hospital of Guangdong Medical University, Zhanjiang, Guangdong,534001, China.

**Keywords:** long non-coding RNA, osteosarcoma, GPx, TrxR, ROS

## Abstract

**Objective:** Most patients with osteosarcoma (OS) have an extremely poor prognosis. The primary purpose of this investigation was to explore the biological effect of Lnc-CLSTN2-1:1 on OS and the potential processes involved.

**Materials and procedures:** We selected differentially overexpressed Lnc-CLSTN2-1:1 from our laboratory's existing RNA sequence analysis data (fibroblast osteoblast (hFOB 1.19) and three osteosarcoma cell lines (HOS, MG63, and U2OS) as the research object. Next, we detected Lnc-CLSTN2-1:1 in the osteosarcoma HOS cell line and fibroblast cells using qRT-PCR. We evaluated cell proliferation ability using EdU incorporation test, CCK-8 test, and cell clone formation; cell invasion and migration were assessed using the Transwell test, while flow cytometry examined cell cycle, apoptosis, and reactive oxygen species (ROS); Subsequently, the activity changes of selenase (GPx) glutathione peroxidase and (TrxR) thioredoxin reductase were detected. In addition, changes in related proteins were analyzed through Western blotting.

**Results:** The expression of Lnc-CLSTN2-1:1 in osteosarcoma cells was significantly increased. The proliferation, invasion, and migration of osteosarcoma cells were significantly inhibited by knockdown of the expression of Lnc-CLSTN2-1:1, and the cell cycle-related signaling pathway PI3K/AKT/GSK-3β/cycinD1 was also inhibited. However, insulin-like growth factor-1 (igf-1) could reverse this process. In addition, we examined the activity of two selenophenases (TrxR and GPx) and the changes of ROS before and after Lnc-CLSTN2-1:1 knockdown. The results showed that both TrxR and GPx activities were reduced after Lnc-CLSTN2-1:1 knockdown, resulting in the inhibition of antioxidant stress levels, while intracellular ROS levels were high, which eventually caused killing effects on tumor cells due to the imbalance between oxidative stress and antioxidant stress.

**Conclusion:** Our results showed that Lnc-CLSTN2-1:1 enhanced anti-oxidative stress TrxR and GPx selenoprotein activities through the PI3K/AKT signaling pathway while counteracting the loss of reactive oxygen species ROS produced by mitochondria to osteosarcoma cells, which protected osteosarcoma cells and thus promoted the proliferation and metastatic ability of OS.

## Introduction

Osteosarcoma (OS) is a malignant bone tumor originating from undifferentiated mesenchymal cells, affecting mostly children and adolescents aged 0-25 years 1. The combination of surgical resection and chemoradiotherapy is the current gold standard for the treatment of osteosarcoma, and the overall survival rate of osteosarcoma patients has increased dramatically. The five-year survival rate following normal therapy ranges from 55-70%, with approximately 90% of patients retaining their limbs 2. However, the five-year survival rate for patients who acquire metastases, despite routine adjuvant chemotherapy and tumor resection, is roughly 5-20% 3-5. Although various molecularly targeted medicines are available, they have not been used effectively to treat osteosarcoma. In addition, the processes underlying the development and progression of osteosarcoma remain unknown 6. Therefore, the search for prospective molecular targets for OS therapy is a significant obstacle.

Long noncoding RNAs (lncRNAs) are RNA transcripts longer than 200 nucleotides that lack protein-coding capability. Numerous studies have suggested that they play crucial roles in many biological functions, ranging from normal development to disease 7, 8. lncRNAs have a crucial function in several disorders through their interaction with nucleic acids and proteins 9-11. lncRNAs are involved in pathological and physiological processes, including angiogenesis, energy consumption, invasion, migration, apoptosis, cycling, and cellular multiplication 12-16. It is now clear that lncRNAs play a critical role in the etiology and progression of osteosarcoma. For example, LncRNA BE503655 inhibits proliferation, invasion, and migration of osteosarcoma through the Wnt/β-catenin pathway 17, whereas Shen et al. 10 discovered that lncRNA KCNQ1OT1 sponges miR-34c-5p and promotes osteosarcoma development by enhancing aerobic glycolysis through ALDOA. Liu et al. 18 discovered that by functioning as a ceRNA for TUSC5, the lncRNA NR 136400 inhibits cell growth and invasion, which is under the control of miR-8081 in osteosarcoma. Lnc-CLSTN2-1:1 is aberrantly expressed in osteosarcoma cells; however, its biological activity has not yet been described. This research aimed to assess the usefulness of Lnc-CLSTN2-1:1 in osteosarcoma and analyze its probable pathways.

Selenases play a key role in maintaining cellular balance owing to the high reactivity of the selenol moiety (-SeH) in Sec. Glutathione peroxidase (GPx) and thioredoxin reductase (TrxR) are well-known redox-active selenates with redox-active sites containing selenol groups 19. Oxidative stress-mediated damage is associated with several pathological conditions such as cancer, rheumatoid arthritis, chronic inflammation, and neurodegenerative diseases 20, 21. Metabolic abnormalities in cancer often lead to oxidative stress and cancer cells maintain REDOX balance by upregulating internal antioxidant systems (such as GPx, TrxR) to protect themselves from increased levels of reactive oxygen species (ROS) and death, thereby maintaining and promoting tumor development 22. Antioxidants can prevent the development of cancer. For example, the gold compound [Au(d2pype)2]Cl, an inhibitor of selenium antioxidant enzymes, induces oxidative stress in lymphoma cell lines, thereby inducing apoptosis and inhibiting the progression of lymphoma 23. Under normal physiological conditions, there is a balance between antioxidant and oxidative stress, which plays an important role in the occurrence and development of tumors. For example, LncRNA GSA5 affects the development of malignant melanoma by regulating the intracellular REDOX balance 24. MicroRNAs develop resistance to chemotherapy and radiotherapy in cancer cells by regulating REDOX balance 25.

ROS are oxygen-containing molecules characterized by their high reactivity and act as oxidants or reducing agents in a wide range of redox reactions 26. Among cancer-causing factors, oxidative stress is essential for tumor development and progression 27. In cancer cells, ROS are aerobic metabolic products of organelles such as mitochondria 28. Maintaining the cellular redox balance is dependent on the balance between ROS generation and ROS consumption by antioxidant systems (e.g., GPx and TrxR) 29-32. ROS is a double-edged sword; at low concentrations, ROS may function as a pro-tumorigenic signaling molecule, while at high ROS concentrations, it may act as a signaling molecule due to its deleterious, genotoxic, and even pro-apoptotic effects on cancer cells and may act as a cancer regulator 33-36. LncRNAs play a role in regulating redox homeostasis in cancer cells by coordinating ROS 37.

## Materials and Methods

### Reagents

IGF-1 was acquired from Apex Biotech (TX, USA), and utilized at a concentration of 200 µg/mL.

### Cell Culture

A human osteosarcoma cell line, HOS, and a human osteoblast cell line, hFOB 1.19, were acquired from the Chinese Academy of Sciences (Shanghai, China) and stored in MEM basal medium and hFOB-specific medium, respectively. 10% serum (zeta life, Z7181FBS-500), penicillin (Baoman Bio, China), and streptomycin (Sigma-Aldrich) were added to all basal media (Baoman Bio, China). Both HOS and hFOB 1.19 cells were kept at 37 ℃ with 5% carbon dioxide in a cell incubator.

### Cell transfection

GenePharma (Shanghai GenePharma, China) produced siRNA. SiNC (NC): positive 5'-UUCUCCGAACGUGUCACGUTT-3', antisense 5'-ACGUGACACGUUCGGAGAATT-3' SiRNA237 (SI1#) sequence: 5'-GCAGCUAUGUUCAGUGUAATT-3', antisense: 5'-UUACACUGAACAUAGCUGCTT-3'; SiRNA2494 (SI2#) sequence: 5'CCAACUUCCUUCGGUUUUTT3', antisense: 5'AAACCGAGAAGGAAGUUGGTT-3'. Following 48 hours of transient transfection with the proper siRNAs (50nM), the cells were harvested for subsequent experiments.

### Solation of RNA with qRT-PCR

TRIzol reagent was used to extract total RNA from the grown cells by the manufacturer's instructions (Vazyme Biotech Co., Ltd, Nanjing, China). Spectrophotometry was used to quantify the isolated RNA using a NanoDrop spectrophotometer (Thermo Fisher Scientific, USA). First-strand cDNA was produced using a cDNA synthesis kit (Takara, Otsu, Japan). The PrimeScript^tm^R reagent Kit (Takara) and ABI Real-time PCR System were used for real-time fluorescence PCR (Applied Biosystems 7500, California, USA). The relative values were calculated using the optimal comparative Ct(2^-ΔΔCt^) value technique following the MIQE standards 38, 39. Three sets of real-time PCR reactions were conducted. GAPDH (forward, 5'-UGACCUCAACUACAUGGUUTT-3'; reverse, 5'-AACCAUGUAGUUGAGGUCATT-3') and Lnc-CLSTN2-1:1 (forward, 5'-ACCTTGGTCCTAGTCCGCATCC-3'; reverse, 5'-AGTGACCTCATGGCAGCCTCTC-3').

### CCK-8 detection experiment

We followed the manufacturer's guidelines using the CCK-8 (ZETA LIFE, K009) cell counting kit. Following transfection with siRNA for 48 h, the density of the cells in 96-well plates was 2,000 human osteosarcoma (HOS) cells per well. Furthermore, 10ul of CCK-8 solution per well was added to the cells, and the cells were treated at 37 °C. Optical density (OD) values were recorded every 24 h at 450 nm during the first 0 h of cell wall formation. Each experiment was conducted in triplicate.

### Experiment with the development of colonies

After 48 h of transfection with siRNA, 500 HOS cells were planted in each well on a plate with a total of six wells and incubated at 37 °C in MEM with 10% fetal bovine serum. The colonies were treated with paraformaldehyde and stained with a crystal violet solution containing 0.1% after seven days. To calculate the number of clones, colonies with more than 50 cells were counted. Each experiment was conducted in triplicate.

### Experiment with EdU integration

The BeyoClickTM EdU-555 Cell Proliferation Kit (C0075S, Beyotime Biotechnology, Shanghai, China) was used to conduct EdU incorporation tests according to the instructions from the maker. OS cells were cultivated in confocal dishes, and after 48 h of transfection treatment, add 200 µl of EdU (0.1mM) was to each well, and the mixture was incubated for 2 h. Immunostaining Wash (P0106, Beyotime Institute Biotech, Haimen, China) was used to fix the cells with 4% paraformaldehyde for 10 min before washing and permeabilizing them for 15 min. After cleaning, the cells were cultured in the dark for 30 minutes adding 0.5 ml of the click response solution, washed, labeled with 1X Hoechst 33342 for 10 min, rinsed, and confocal microscopy pictures were acquired (Zeiss). Three separate tests were conducted.

### Migration and invasion assay in Transwell

The HOS cells were harvested 48 hours after siRNA transfection. 20000 cells were injected into the top chamber of a 24-well plate in 200 µl of serum-free medium (COStar Inc., USA). For the invasion experiment, Matrigel (Corning) at a density of 250 g/ml was used to coat the top chamber, which was then heated to 37 °C for 2 h. The top chamber was then injected with 20000 cells suspended in 200 µl of serum-free media. The bottom chamber of the transwell was filled with 600 µl of media containing 10% fetal bovine serum to permit cell migration. After 24-48 hours of cell growth, the top membrane was wiped using a cotton swab to remove any remaining cells. Following transcytosis, the cells were fixed with 4 percent paraformaldehyde and stained with 0.1% crystal violet. In each chamber, an inverted microscope (Olympus, Tokyo, Japan) was used to take photographs of five random fields and tally the results.

### Flow cytometry investigation of the cell cycle and apoptosis

Forty-eight hours after siRNA transfection, the HOS cell lines were removed and cleaned with PBS. The cells were set in an ethanol solution of 70% strength and had been pre-chilled before being stored at 4 °C overnight. The next day, the cells were rinsed with PBS and cultured for 30 minutes in the dark. using the Cell Cycle Check Kit (Beyoncé C1052). Then, a flow cytometer and Modfit software were used to study HOS cells (BD Biosciences, USA), determining the total number of cells in the G0/G1, S, and G2/M phases and comparing their percentages.

Apoptosis was identified using an Apoptosis Detection Kit (BD PE Annexin V Apoptosis Detection Kit l, USA). After 48 h of transfection, the cells with phosphate buffer (PBS). Following this step, 1 × 106 cells/ml were resuspended in 1X binding buffer. After transferring 100 µl of the solution (1 × 105 cells) to a loss tube, 5µl 7-AAD, and PE-annexin V were added. The cells were gently mixed before being placed in an incubator for 15-20 minutes at ambient temperature and complete obscurity. Following that, the cells were cultured for 15 minutes at room temperature and completely obscured. After 400 µl of 1X binding buffer was added at each time interval. Flow cytometry (FACScan, Becton-Dickinson) was used to assess the samples. PE-annexin V-/7-AAD -, PE-annexin V+/7-AAD, and PE-annexin V+/7-AAD - signals were observed in live, late, and early apoptotic cells, respectively. Each experiment was performed in triplicate.

### Cytosolic thioredoxin reductase (TrxR) activity and glutathione peroxidase (GPx) activity assay

Next, 3*10x5/well HOS cells were grown in a six-well plate and after 72 h of cell transfection, proteins were extracted and assayed using Thioredoxin Reductase (TrxR) activity kit (BC1155, Solarbio Science & Technology, Beijing, China). A 96-well plate was taken and put for about 30 min at 37°C, protected from light, after adding the corresponding reagents. A412 was measured using an enzyme marker, and the TRXR activity was calculated according to the formula for the 96-well plate assay according to the reagent instructions. For the Glutathione Peroxidase Assay (DTNB) (S0057S, Beyotime Biotechnology, Shanghai, China), a 96-well plate was taken, and the corresponding solutions according to the instructions were added, mixed well, and incubated for 10 min in the dark at room temperature. Then, each well received 6.6 µL of the DTNB solution, which was well mixed in. After incubation for 10 min, A412 was measured using an enzyme marker, and glutathione oxidase activity was calculated according to the formula in the reagent instructions.

### Detection of thioredoxin reductase (TrxR) activity and glutathione peroxidase (GPx) activity in animal tissues

Tissue was homogenized in an ice bath at a ratio of 1:5-10 tissue mass (g): reagent volume (mL) using a Thioredoxin Reductase (TrxR) activity assay kit (BC1155, Solarbio Science & Technology, Beijing, China) by the manufacturer's guidelines. The supernatant was removed after centrifugation at 10,000 rpm at 4 °C for ten minutes. A 96-well plate was taken, and the corresponding reagents were added, and incubated for 30 min at 37℃, avoiding light. Then an enzyme marker was used to determine A412 and calculate the TRXR activity according to the formula for 96-well plate determination in the reagent instructions. For the Glutathione Peroxidase Assay Kit (DTNB method) (S0057S, Beyotime Biotechnology, Shanghai, China), tissue samples were obtained after the animals were cleared of blood using saline perfusion. The samples were homogenized in a glass homogenizer in an ice bath at 4 °C and 12,000 rpm for approximately 10 minutes, at a ratio of approximately 200 µL of sample homogenate to 20 mg of tissue, and the supernatant was removed. To a 96-well plate, the corresponding solutions in order according to the instructions were added, mixed well, and incubated at room temperature for ten minutes; then 6.6 μL of DTNB solution was put into every hole and mixed well. After incubation at room temperature for 10 min, A412 was measured using an enzyme marker, and glutathione oxidase activity was calculated using the formula provided in the manufacturer's instructions.

### Reactive Oxygen Specimen (ROS) detection

The kit for measuring reactive oxygen species (C0075S, Beyotime Biotechnology, Shanghai, China) was used according to the guidelines provided by the producer 48 h after transfection of HOS cells with siRNA and washed with PBS. Each 6-well plate sample received 1 mL of diluted DCFH-DA after the medium was removed. DCFH-DA (10 mol/L) was diluted to 1:1000 in a basic medium. The cells were then incubated in a cell culture incubator for 30 min. Cells were thoroughly cleaned with basic media 3 times to remove DCFH-DA that had not been incorporated into the cells. The cells were collected and analyzed using flow cytometry.

### Western blotting

72 hours after transfection, Cells from an OS were gathered and put into a RIPA buffer (Solarbio, Beijing, China). The samples were separated using SDS-PAGE (Solarbio, Beijing, China) for two hours at 120V before being moved to PVDF (Millipore, Billerica, MA) membranes. After blocking the membranes in 5% skim milk for 2 h, they were incubated overnight at 4 °C with the first antibody and then incubated in the secondary antibody solution for 1 hour, and the immunoreactive bands were detected using chemiluminescence (Tanon 5200, Shanghai, China). Anti-IP3K, p-IP3K, AKT, p-AKT, GSK-3β, p- GSK-3β, cyclinD1, and ki67 monoclonal antibodies produced by a rabbit [Cell Signaling Technology (CST), Inc., Danvers, MA, USA] and anti-actin antibodies from mice were diluted 1:1000 with the first antibody. Secondary antibodies (rabbit anti-mouse IgG/HRP) were diluted 1:5000 in TBST [Cell Signaling Technology (CST), Inc., Danvers, MA, USA]. The intensities of the target protein bands were standardized relative to the intensities of the β-actin bands. The image-ProPlus program evaluated grayscale values (Cybernetics, Inc., USA).

### Mouse xenograft model

The investigations were conducted on BALB/C nude mice (female, 4 weeks old) after one week of upbringing, and every animal experiment was conducted according to the protocol for animal care approved by Guangdong Medical University's Animal Care Committee. Firstly, a total of 9 mice were randomly divided into 3 groups, with 3 mice in each group, and all animals were injected subcutaneously into the left axilla with 1.0x10^6^ premixed stromal gel target cells. Every 4 days the tumor size was documented. After 28 days, every mouse was sacrificed and the tumors were isolated. The volume and mass of each extracted xenograft tumor were quantified. Tissue samples were subsequently preserved and processed for further histological investigation, and animal experiments were conducted with the ethics committee's approval (No. Approved: AHGDMU-LAC-IV(1)-2207-B003) and following national or institution-specific guidelines for animal care and use.

### Analytical statistics

All cell function investigations were conducted three times to ensure repeatable findings. The results of the three different studies are presented as the mean standard deviation error (SE). Statistical analysis and graphical representation were executed in GraphPad Prism 9. (GraphPad, CA). Statistical significance was assumed at a level of 0.05.

## Results

### Expression of lncRNAs in osteosarcoma

**Figure 1:**
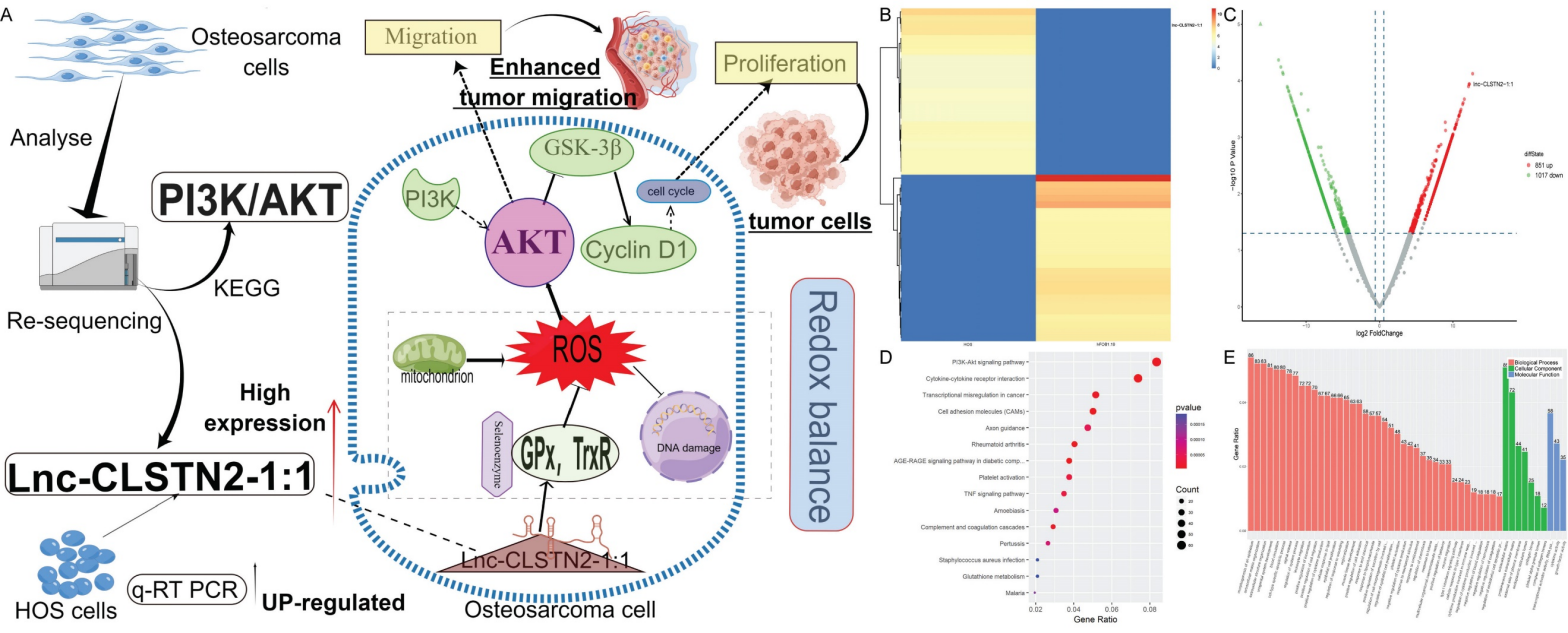
Patterns of gene expression that are different between osteosarcoma (OS) cells and osteoblasts, and analysis of KEGG and GO for lncRNAs and mRNAs that are expressed differently. (A, by figdraw) Schematic and mechanism of Lnc-CLSTN2-1:1 overexpression in osteosarcoma cells and promotion of proliferation, invasion, and migration through disruption of intracellular REDOX balance. (B) cluster analysis reveals the presence of lncRNA files. Yellow denotes high levels of expression, whereas blue shows low levels of expression. In the heat map, each sample is shown as a column, and each lncRNA is shown as a row. (C) Differentially expressed lncRNAs were separated using a volcano plot. The vertical lines show 1.5-fold changes, whereas the horizontal lines indicate 0.05 significance levels. (D) Analysis of pathways based on the KEGG database. (E) Results based on an examination of GO enrichment.

Total RNA sequencing results of osteosarcoma cell line HOS and human osteoblast hFOB 1.19 were used to cluster differentially expressed lncRNA according to hierarchy (Fig. 1B). The lncRNA expression profile revealed the existence of 1868 differentially expressed lncRNAs after data filtering (fold change ≥2.0, P value <0.05), including 851 upregulated lncRNAs and 1017 downregulated lncRNAs (Fig. 1C). The differentially expressed lncRNAs and mRNAs were connected to 15 distinct signaling pathways, as determined by KEGG pathway enrichment analysis. One of the most crucial routes involved was called "PI3K/AKT" (Fig. 1D). GO enrichment biological process: promotion of cell migration (Fig. 1E). From the group of upregulated lncRNAs, we selected Lnc-CLSTN2-1:1 (FDR< 0.05, fold change >1.5, and p < 0.05) to investigate their biological roles in osteosarcoma.

### Lnc-CLSTN2-1:1 is increased in OS and knocking it down reduces the development of OS cells

**Figure 2:**
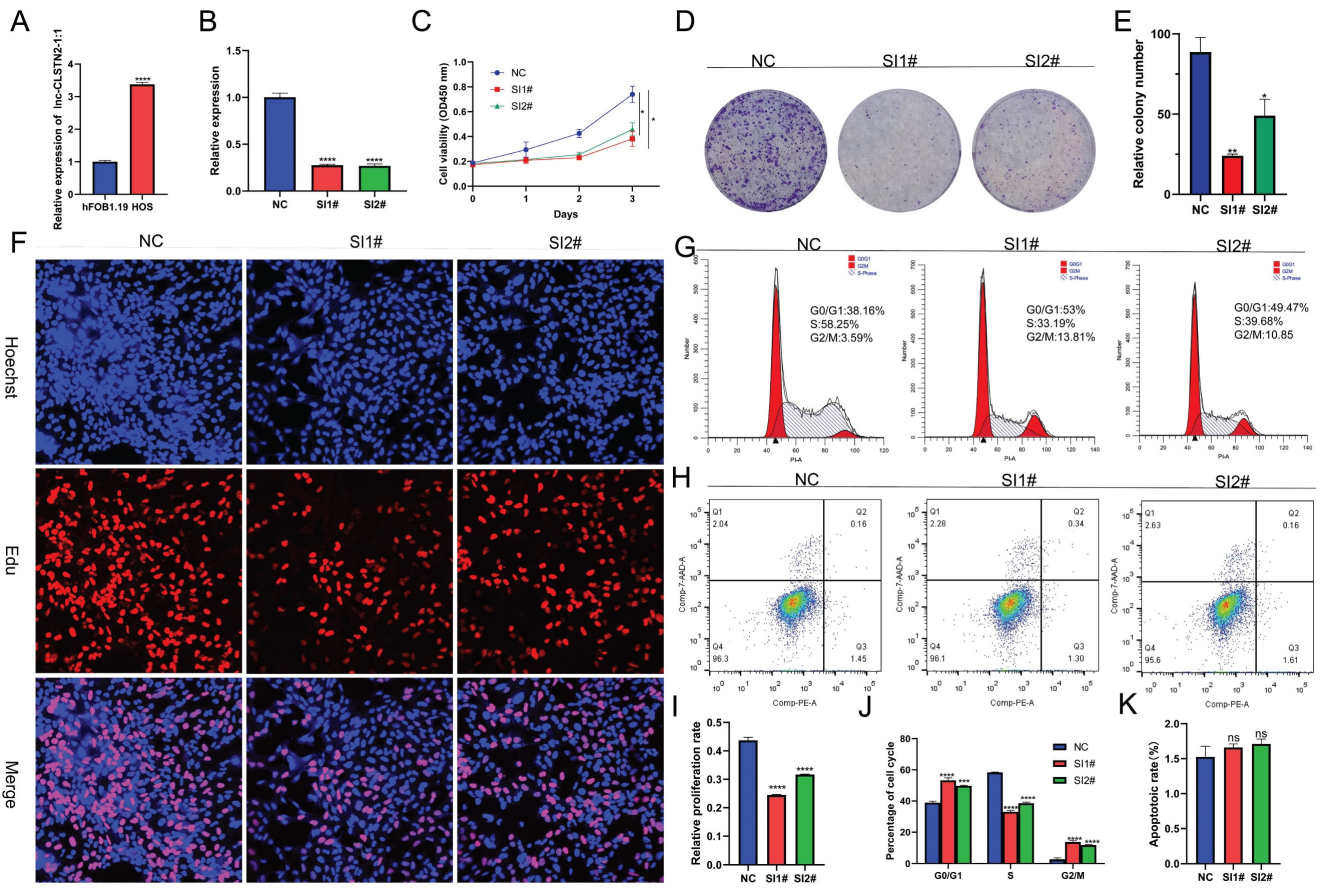
Effect of Lnc-CLSTN2-1:1 knockdown on in vitro OS cell viability. (A) Expression levels of Lnc-CLSTN2-1:1 was decided by qRT-PCR in osteosarcoma HOS cells and osteoblasts. (B) Lnc-CLSTN2-1:1 expression levels show knockdown effectiveness in HOS cells injected with siRNA (C) After Lnc-CLSTN2-1:1 knockdown, CCK-8 was used to count the number of living HOS cells. (D, E) Formation of colonies in HOS cells after Lnc-CLSTN2-1:1 knockdown. (F, I) EdU incorporation experiments showed that the proliferation capacity of Lnc-CLSTN2-1:1 knockdown HOS cells was decreased compared with control cells. (G, J) Flow cytometry analysis showed the cycle changes of HOS cells after Lnc-CLSTN2-1:1 knockdown. (H, K) Flow cytometry further detects apoptosis by doubly labeling cells with PE-annexin V and 7-AAD. The results are shown in bar graphs as mean standard error (n = 3) (*P < 0.05, **P < 0.01, ***P < 0.001, ****P < 0.0001, similarly hereinafter).

Lnc-CLSTN2-1:1 was further verified as an upregulated lncRNA using qRT-PCR in HOS cells to validate the RNA-Seq findings. Consistent with the RNA-Seq findings, Inc-CLSTN2-1:1 was considerably elevated in OS tissues relative to the normal line of human osteoblast cells (hFOB1.19) (Fig. 2A). To examine the biological function of Lnc-CLSTN2-1:1 in OS development, we generated two siRNAs (SI1# and SI2#) and a control (NC), and transfected HOS cell lines with Lnc-CLSTN2-1:1-specific siRNA. The knockdown effectiveness was assessed by qRT-PCR (Fig. 2B). As lncRNAs are engaged in a wide range of biological processes, we decided to determine the effect of Lnc-CLSTN2-1:1 on OS cells. The Cell Counting Kit-8 (CCK-8) test discovered that Lnc-CLSTN2-1:1 knockdown decreased HOS cell growth (Fig. 2C). The role of Lnc-CLSTN2-1:1 in long-term survival was tested by colony formation assays. (Fig. 2D, E) demonstrates that the knockdown of Inc-CLSTN2-1:1 diminished the population's capacity to form colonies. EdU experiments (Fig. 2F, I) revealed that silencing Lnc-CLSTN2-1:1 substantially hindered HOS cell proliferation.

The cell cycle and apoptosis are essential biological processes involved in cell development. FACS technology was used to assess whether the effect of Lnc-CLSTN2-1:1 on osteosarcoma cell proliferation was due to Lnc-CLSTN2-1:1-mediated alterations in the cell cycle or apoptotic mechanisms. Relative to control cells, After Lnc-CLSTN2-1:1 was inhibited, increased number of cells in the G0/G1 phase, while the number of S phase cells reduced (Fig. 2G, J). Nevertheless, using flow cytometry, we assessed the effect of Lnc-CLSTN2-1:1 on apoptosis. There was no discernible increase in apoptosis in the treatment group versus the control group in HOS cells exposed to SI1# or SI2# (Fig. 2H, K). Therefore, we hypothesized that low Lnc-CLSTN2-1:1 expression inhibits cell growth in OS cell lines via cell cycle arrest rather than death.

### Lnc-CLSTN2-1:1 expression suppression in OS cells impairs invasion and migration

**Figure 3:**
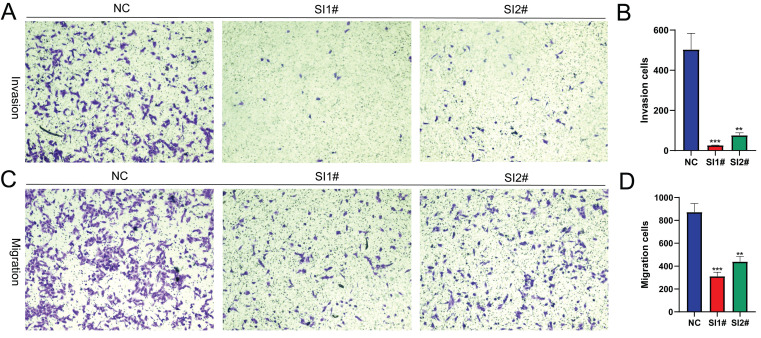
Implication of Lnc-CLSTN2-1:1 silencing in the invasion and migration of in vitro OS cells. Transwell assay was performed on HOS cells transfected with NC, SI1#, or SI2#. (A, B) were the results of the invasion assay and (C, D) were the results of the migration assay. All results were replicated 3 times. Data are presented as mean ± SE.

Cell invasion and migration as the biological behavior of cancer metastasis, to find out how Lnc-CLSTN2-1:1 influences the invasion and migration of cancer cells, HOS cells were used in transwell experiments. Compared to the control group, Lnc-CLSTN2-1:1 downregulation caused a significant drop in the number of cells that invaded and moved around (Fig. 3A-D). These results show that Lnc-CLSTN2-1:1 may be part of a process linked to the OS's ability to spread to other parts of the body.

### Knockdown of Lnc-CLSTN2-1:1 inhibits selenate activity and leads to the accumulation of ROS

**Figure 4:**
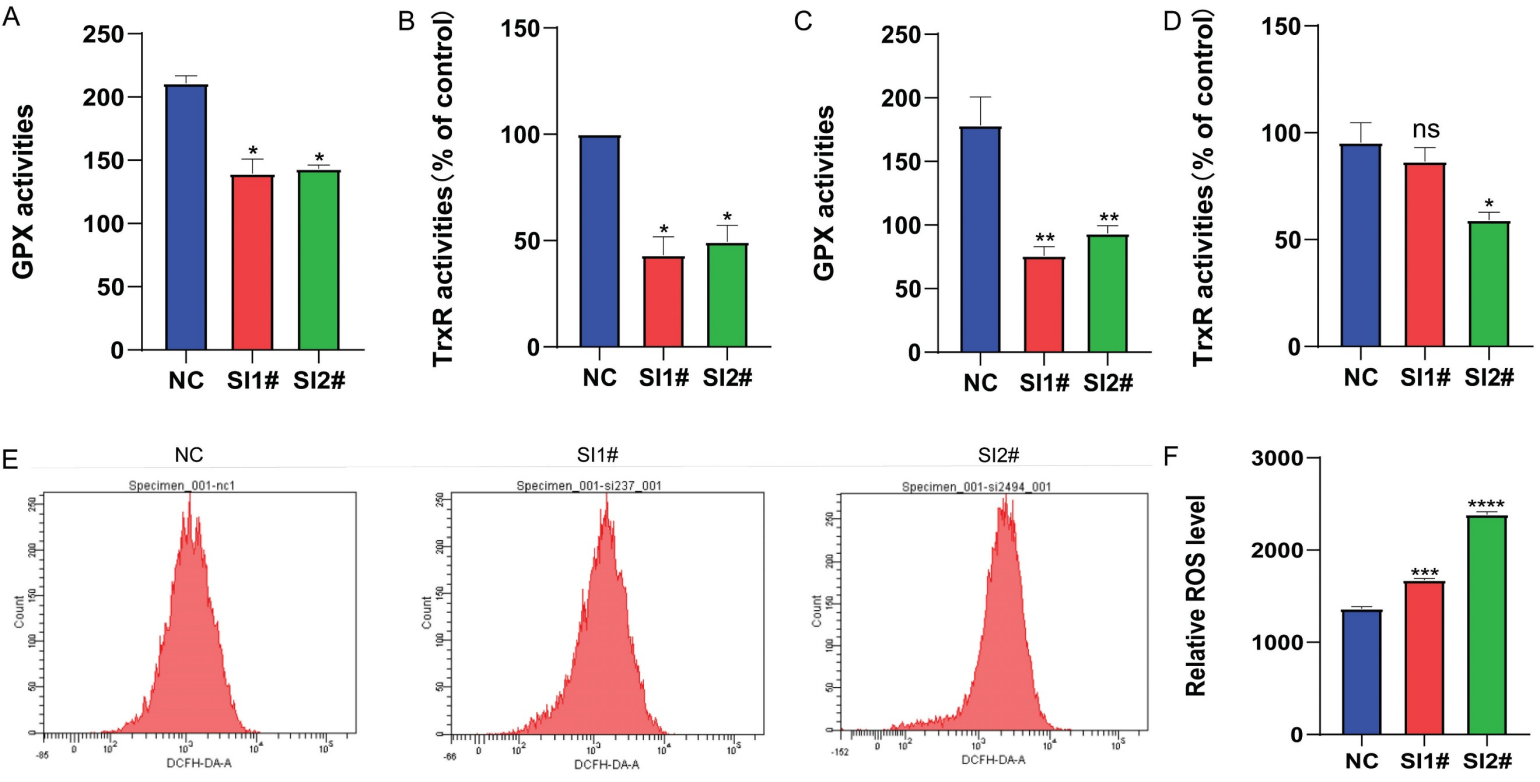
Changes in selenate and ROS after Lnc-CLSTN2-1:1 knockdown treatment. (A, B) Changes in selenoenzyme (GPx, TrxR) activity were detected after the knockdown of Lnc-CLSTN2-1:1 in the osteosarcoma cell line HOS. (C, D) Changes in selenase (GPx, TrxR) activity were detected in tumor-forming tissues of nude mice. (E, F) Detection of ROS levels after knockdown of Lnc-CLSTN2-1:1 in the osteosarcoma cell line HOS. Averages and standard errors are shown for the data.

Redox alterations can change the fate of cancer cells, and whether Lnc-CLSTN2-1:1, a regulatory gene with no self-encoding ability, affects the redox balance within cancer cells to promote cancer development. Therefore, we examined the changes in the pro-oxidant reactive oxygen species (ROS) antioxidant system selenate (GPx, TrxR). The results showed that GPx and TrxR expression was reduced in osteosarcoma hos cells after siRNA knockdown, with TrxR being more significantly reduced (Fig. 4A, B). In nude mice, the expression of GPx and TrxR was lowered in the assayed siRNA group compared to the unprocessed group, but the activity of TrxR was not statistically significant in SI1# (Fig. 4C, D). After 24 h of siRNA knockdown therapy, reactive oxygen species levels in osteosarcoma hos cells were considerably greater than those in the control group, thus leading to higher ROS accumulation in the cells and further depletion of the antioxidant system selenate (GPx, TrxR) (Fig. 4E, F), due to excessive ROS levels leading to damage of some very important components of cells such as some macromolecular lipids, nucleic acids, and proteins, which eventually lead to cell death 40 for tumor cells. These results show that: After the expression of Lnc-CLSTN2-1:1 was knocked down in osteosarcoma cells by RNAi technology, the in vivo and in vitro experimental results suggested that the expression level of antioxidant system selenate (GPx, TrxR) was decreased, while the expression level of ROS was increased, destroying REDOX balance and the damage of ROS to cancer cells. Slowed the progression of osteosarcoma.

### Lnc-CLSTN2-1:1 promotes OS progression through activation of the PI3K/AKT/GSK-3β/cyclinD signaling axis

**Figure 5:**
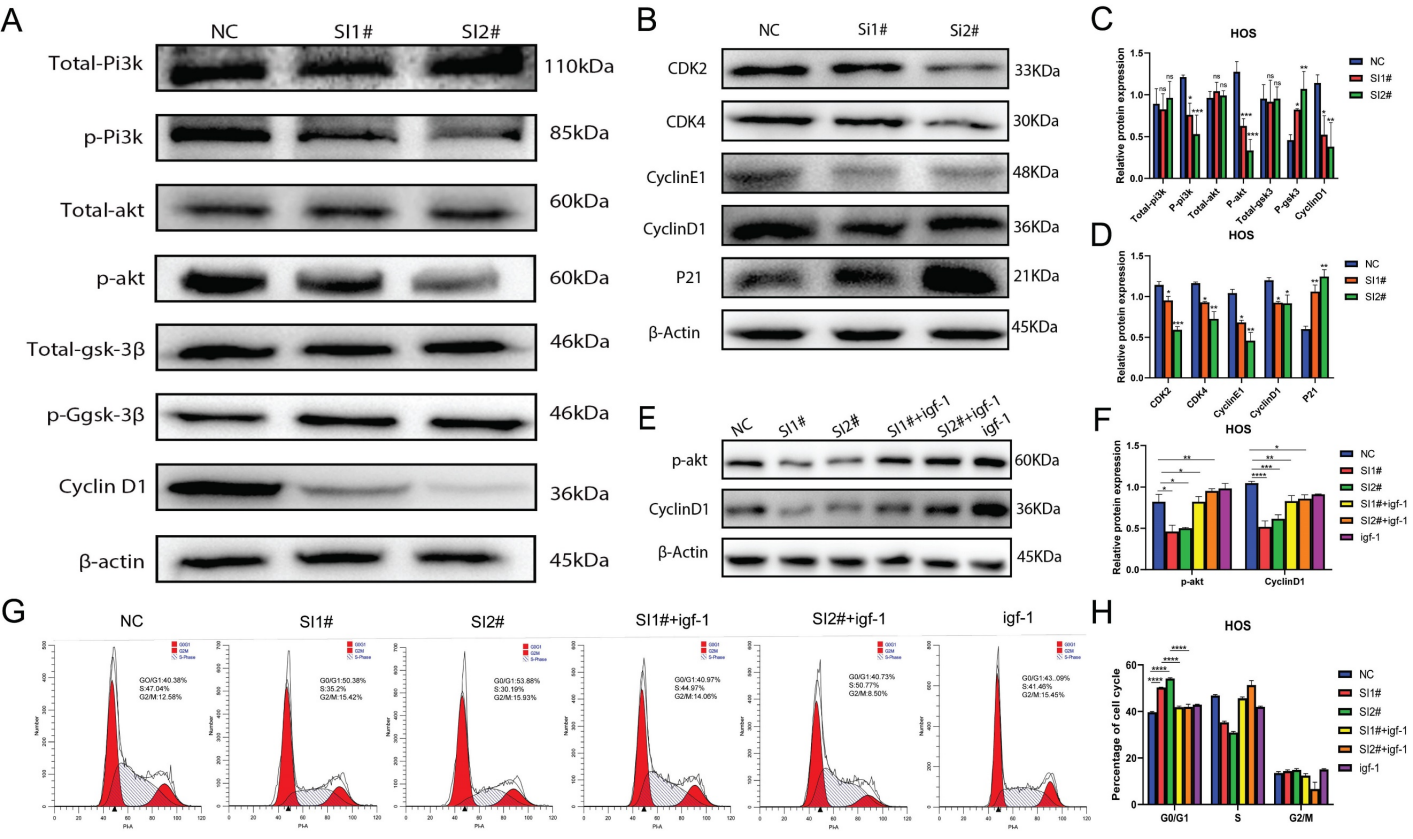
Lnc-CLSTN2-1:1 increases the development of OS through the PI3K/AKT/GSK-3β/cyclinD1 axis. (A, C) In HOS of Lnc-CLSTN2-1:1 knockdown OS cell lines, the ranges of phosphorylated p-PI3K, p-AKT, cyclinD1, and p-GSK-3β were lowered, although the total of PI3K, AKT, and GSK-3βwere unaltered. (B, D) The CDK2, CDK4, cyclinE1, and cyclinD1 checkpoint proteins of the G0/G1 phase of the cell cycle were down-regulated, but the P21 protein was up-regulated. (E, F) For one hour, igf-1 was used to pre-treat the HOS cells and then transfected with siRNA while maintaining a consistent level of igf-1 in the igf-1 group. The expression of P-AKT and cyclinD1 was then evaluated using a protein blot assay. (G, H) A flow cytometer was utilized to track the cell cycle. The internal reference protein was β-Actin. The bar graph depicts the normalized protein concentrations of HOS cells. Averages and standard errors are shown for the data.

According to prior enrichment analysis, KEGG determined that the primary importance for controlling cell proliferation, invasion, and migration is the PI3K/AKT signaling system. The PI3K/AKT signaling pathway is widely acknowledged to play a pivotal role in controlling cell proliferation, invasion, and migration. Consequently, we examined whether Lnc-CLSTN2-1:1 also modulates the PI3K/AKT pathway. To better understand the molecular process by which Lnc-CLSTN2-1:1 enhances OS cell proliferation, invasion, and migration, we performed protein blotting to identify cyclin D1 (cyclinD1), a cell cycle protein that regulates G0/G1 phase cell arrest. CyclinD1 levels decreased in HOS cells conjugated with SI1 and SI2 (Fig. 5A). While cyclinD1 upstream molecules were phosphorylated in the Lnc-CLSTN2-1:1 knockdown OS cell line HOS, decreased protein levels of p-IP3K, p-AKT, p-GSK-3β, total IP3K, AKT, and GSK-3β were maintained (Fig. 5A, C). These findings imply that Lnc-CLSTN2-1:1 regulates osteosarcoma development through the PI3K/AKT/GSK-3 β/cyclin D signaling axis. This signaling pathway is a cell cycle-related pathway, and we then examined the cyclins associated with the G0/G1 checkpoint: CDK2, CDK4, cyclinE1, and cyclinD1 are down-regulated, while P21 proteins are up-regulated after knockdown therapy, which is consistent with flow cytometry results (Fig. 5B, D). To determine the impact of Lnc-CLSTN2-1:1 on the cell cycle via the PI3K/AKT pathway, which contributes to the malignant biological behavior of osteosarcoma, we pretreated cells with igf-1 (PI3K /Akt agonist) and then measured the protein levels of P-AKT and cyclinD1. The levels of P-AKT and cyclinD1 were considerably higher in the igf-1-treated group than in the siRNA-treated group (Fig. 5E, F). Periodic results revealed that the GO/G1 phase of the cell cycle was considerably reversed in igf-1-treated cells relative to siRNA-treated cells (Fig. 5G, H).

### The inhibition of Lnc-CLSTN2-1:1 expression inhibits the development of tumors in vivo

**Figure 6:**
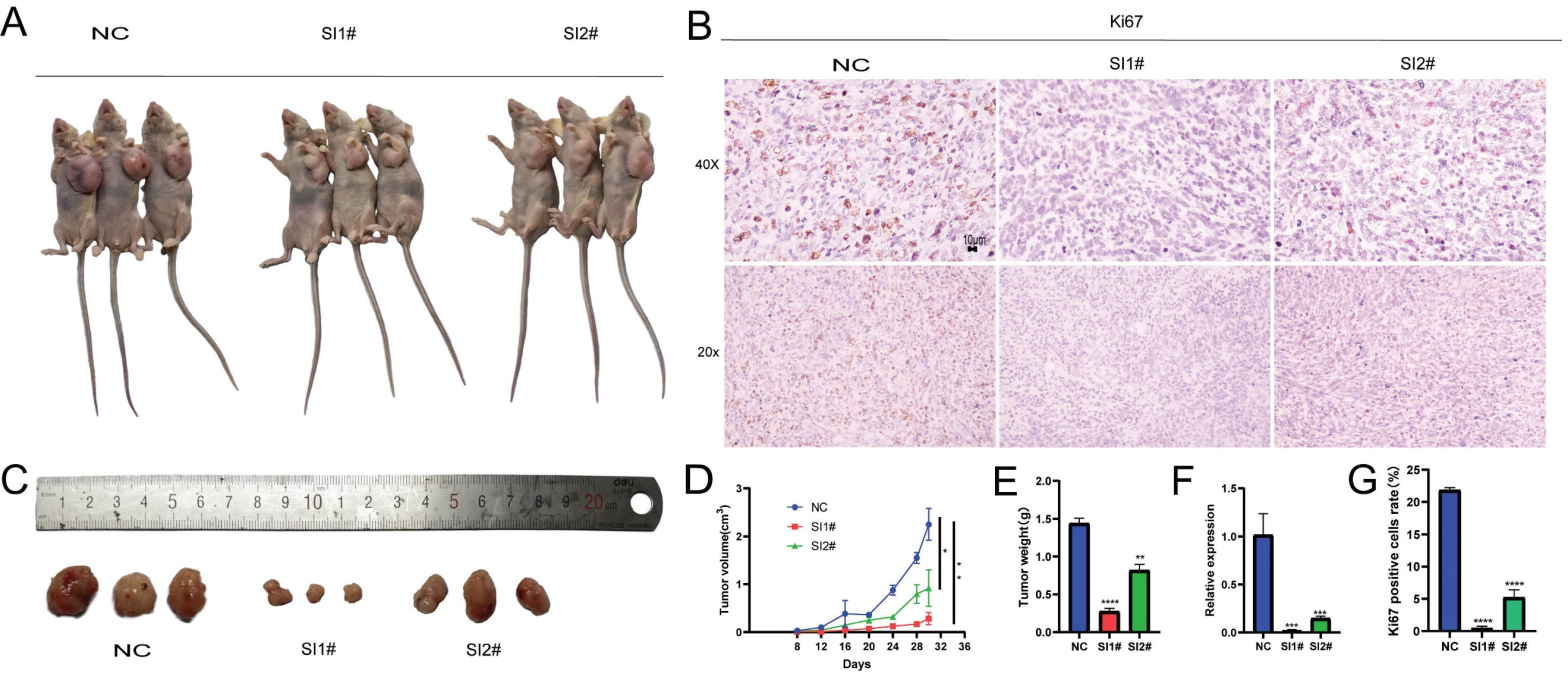
Illustration of the effect of Lnc-CLSTN2-1:1 downregulation on tumor development in vivo.After injection of NC, SI1#, and SI2# transfected HOS cells, tumor growth curves were evaluated, and tumor sizes were determined every four days (A, D). Tumor weights at the time of harvest (C, E). (F) qRT-PCR analysis of Lnc-CLSTN2-1:1 expression levels in tumor tissues resulting from NC, SI1#, and SI2# transfections of HOS cells. (B, G) Representative Ki-67 immunohistochemical images of the tumors. Immunostaining: Scale bars denote 10 µm. Data are presented as mean ± SE.

After analyzing the biological effects of Lnc-CLSTN2-1:1 using in vitro assays, we present in vitro evidence supporting the oncogenic impact of Lnc-CLSTN2-1:1 in osteosarcoma. To determine whether Lnc-CLSTN2-1:1 has the same effect in vivo, we assessed the effect of Lnc-CLSTN2-1:1 on tumor development using tumor xenografts in nude mice. A xenograft mouse model devoid of HOS cells was created. Subcutaneous injections of a stromal gel mixture containing transfected HOS cells (NC, SI1#, or SI2#) were injected into the naked mice's left axilla. (n = 3 per group). After 28 days after inoculation, Lnc-CLSTN2-1:1 knockdown dramatically decreased tumor development relative to that in the control group (Fig. 6A, D). Additionally, the tumor weight on day 28 was considerably lower in mice transfected with Lnc-CLSTN2-1:1 than in the control group (Fig. 6C, E). Additionally, qRT-PCR showed that Lnc-CLSTN2-1:1 expression in the transfected tumors was lower in the experimental group than in the control group (Fig. 6F). Ki-67 staining was also conducted to confirm these findings (Fig. 6B, G). Lnc-CLSTN2-1:1 knockdown decreased tumor development in vivo, according to the data obtained. These findings suggest that Lnc-CLSTN2-1:1 regulates OS cells in an oncogenic manner.

## Discussion

LncRNAs have been shown to play crucial roles in a variety of human activities, both physiological and pathological 10, 41, 42. The expression of lncRNAs is one of the most common transcriptional changes in cancer in recent genome-wide characterizations of human cancer transcriptomes 43, and a substantial amount of experimental data implies that lncRNAs may be playing a significant role in carcinogenesis 44-46; however, the underlying biological functions and potential mechanistic details of the majority of lncRNAs in human OS remain unknown. According to this study, Lnc-CLSTN2-1:1 was considerably elevated in OS compared to that in normal osteoblasts. Using the knockdown test CCK8, clone creation, EdU assay, and transwell assay, we found that Lnc-CLSTN2-1:1 significantly contributed to the progression of osteosarcoma in terms of value-added (Fig. 2), invasion, and migration (Fig. 3). Moreover, we established that Lnc-CLSTN2-1:1 promotes OS cell growth in an *in vitro* nude mouse carcinogenesis experiment (Fig. 6). These results implied that Lnc-CLSTN2-1:1 may function as an oncogene in OS.

Next, we examined the changes in selenate (GPx and TrxR) against oxidative stress at the cellular and tissue levels, and the results showed that GPx and TrxR expression was reduced in osteosarcoma HOS cells after treatment with siRNA knockdown, with TrxR being more significantly reduced (Fig. 4). In animal tumor-forming tissues, the expression of GPx and TrxR was decreased in the detection siRNA group compared to the non-treated group, but among them, SI1# in TrxR was not statistically significant. In contrast, the level of oxidized ROS was significantly higher in HOS cells after Lnc-CLSTN2-1:1 knockdown than that in the regular group (Fig. 4). These results suggest that the redox balance in osteosarcoma cells is disrupted, prompting higher ROS levels to inhibit osteosarcoma progression. Flow cytometry assay experiments indicated that Lnc-CLSTN2-1:1-induced proliferation of OS cells may be associated with a cell cycle shift from G0/G1 to the S phase. The identification of cell cycle-related proteins (e.g., cyclins, CDKs, and CKIs) is essential for cell cycle control. This regulation stems from the presence of quality control (checkpoints), which ensure that each step of the cell division cycle is completed before the next step occurs 47, 48. In the present study, Lnc-CLSTN2-1:1 knockdown inhibited not only cyclin D1 expression but also CDK4 expression and upregulated P21, thereby suppressing cell cycle suppression in the G0/G1 phase (Fig. 5). However, the turnover of D-type cyclins depends on PI3K and Akt, which negatively regulate the phosphorylation of cyclin D1 on a single threonine residue (Thr-286) *via* GSK-3β^49^. We examined the phosphorylation of PI3K, p-PI3K, AKT, P-AKT, P-GSK-3β, GSK-3β, and cyclinD1 cycle-related pathway proteins by protein blotting assays (Fig. 5). Moreover, after treatment of the cells with igf-1, the downstream proteins P-akt and cyclinD1 were reverted, and the same results were obtained for the blockage of the G0/G1 phase in the flow check results (Fig. 5). This suggests that Lnc-CLSTN2-1:1 knockdown affects the proliferation, invasion, and migration of OS cells by inhibiting the cell cycle through the PI3K/AKT/GSK-3β/cyclinD1 signaling pathway. The changes in antioxidant stress levels (such as GPx and trxr) and ROS levels in tumors are related to the changes in the pi3k/Akt signaling pathway 50-53. Our study found that the expression level of long non-coding RNA (lncRNA) Lnc-CLSTN2-1:1 is elevated in osteosarcoma and promotes the progression of osteosarcoma by interfering with intracellular REDOX balance. Specifically, Lnc-CLSTN2-1:1 causes excessive production of reactive oxygen species (ROS) within cells by activating the PI3K/AKT signaling pathway, thus disrupting the REDOX balance within cells. REDOX balance plays an important regulatory role in cells. Normally, there is a state of equilibrium within cells, that is, a balance between oxidative stress and antioxidants. In cancer, however, this balance is often upset, resulting in the production of too much ROS within cells. Excessive accumulation of ROS triggers oxidative stress within cells, leading to damage to DNA, proteins, and lipids, which promotes the development and progression of cancer. This study found that Lnc-CLSTN2-1:1 increased the production of ROS by activating the PI3K/AKT signaling pathway. The PI3K/AKT signaling pathway is an important cell survival and proliferation signaling pathway that plays a key role in a variety of cancers. By activating the PI3K/AKT signaling pathway, Lnc-CLSTN2-1:1 promoted the proliferation and metastasis of osteosarcoma cells and led to excessive accumulation of ROS. This discovery sheds light on the specific mechanisms of REDOX regulation in cancer. It shows that by regulating the REDOX balance, it is possible to influence the survival and proliferation ability of cancer cells. Therefore, further research on the mechanism of REDOX regulation in cancer will help to develop new therapeutic strategies to improve cancer prognosis and treatment outcomes.

Compared with other LncRNAs associated with osteosarcoma. The biological function and mechanism of action of Lnc-CLSTN2-1:1 in osteosarcoma have not been described. Based on the regulatory role of non-coding RNA (lncRNA) and the balance between oxidative stress and anti-oxidative stress in the tumor microenvironment, we further elaborated the manifestation and mechanism of malignant proliferation, invasion, and metastasis of osteosarcoma. This is a new direction for the study of osteosarcoma and may be a new opportunity for clinical treatment conversion.

The limitations and future directions of this study mainly include the following aspects: 1. Sample size and source: The sample size used in this study was relatively small, including only one human osteosarcoma cell line and one human osteoblast cell line. Future studies could expand the sample size and include cell lines and clinical samples from more sources to increase the reliability and generalization of the study. 2. LncRNA regulated the changes of Glutathione peroxidase (GPx) and thioredoxin reductase (TrxR), which may be related to the regulation of selenol groups or selenium. For example, Selenium plays a role in antioxidants by regulating the activity of peroxidase in antioxidants 54, which needs further verification. 3. Mechanism exploration: Although this study found that the increased expression of Lnc-CLSTN2-1:1 in osteosarcoma cells is associated with cell proliferation, invasion, and migration, as well as the mechanism of regulating antioxidant stress through the PI3K/AKT signaling pathway, the specific molecular mechanism has not been fully clarified. Future studies could further explore the interactions between Lnc-CLSTN2-1:1 and other molecules, as well as its specific mechanisms of action in cell signaling and regulatory networks. 4. Clinical transformation: Although this study has preliminarily explored the role of Lnc-CLSTN2-1:1 in osteosarcoma, its clinical application prospects are still unclear. Future studies could further investigate the expression level of Lnc-CLSTN2-1:1 in clinical samples and explore its clinical application value as a potential diagnostic marker or therapeutic target for osteosarcoma. In summary, future studies can further expand the number and sources of samples, further explore the mechanism of action of Lnc-CLSTN2-1:1, and study its clinical application prospects, to promote the further development and clinical transformation of this field.
